# Anticancer Properties of Lobetyolin, an Essential Component of Radix Codonopsis (Dangshen)

**DOI:** 10.1007/s13659-020-00283-9

**Published:** 2020-11-07

**Authors:** Christian Bailly

**Affiliations:** OncoWitan, 59290 Lille (Wasquehal), France

**Keywords:** Cancer therapy, Natural products, Mechanism of action, Molecular target, Terpenoids, Lobetyolin, Polyacetylene glycoside, Glutamine metabolism

## Abstract

Lobetyolin (LBT) is a polyacetylene glycoside found in diverse medicinal plants but mainly isolated from the roots of *Codonopsis pilosula*, known as Radix Codonopsis or Dangshen. Twelve traditional Chinese medicinal preparations containing Radix Codonopsis were identified; they are generally used to tonify spleen and lung Qi and occasionally to treat cancer. Here we have reviewed the anticancer properties of *Codonopsis* extracts, LBT and structural analogs. Lobetyolin and lobetyolinin are the mono- and bis-glucosylated forms of the polyacetylenic compound lobetyol. Lobetyol and LBT have shown activities against several types of cancer (notably gastric cancer) and we examined the molecular basis of their activity. A down-regulation of glutamine metabolism by LBT has been evidenced, contributing to drug-induced apoptosis and tumor growth inhibition. LBT markedly reduces both mRNA and protein expression of the amino acid transporter Alanine-Serine-Cysteine Transporter 2 (ASCT2). Other potential targets are proposed here, based on the structural analogy with other anticancer compounds. LBT and related polyacetylene glycosides should be further considered as potential anticancer agents, but more work is needed to evaluate their efficacy, toxicity, and risk–benefit ratio.

## Introduction

**Fig. 1:**
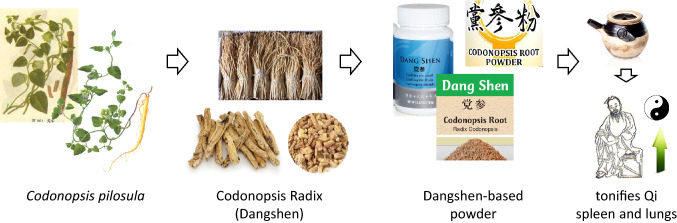
*Codonopsis*  *radix* is made from the dried roots of the plant *Codonopsis pilulosa*. The plant is generally cultivated. In TCM, Dangshen bulk herb is used in the daily dosage of 9 to 30 g. In most cases, bulk herbs are cooked in boiling water to make tea or soup for consumption. The use of Dangshen invigorates lung and spleen qi, nourishes the blood, and generates body fluids

Radix Codonopsis or Dangshen in Chinese (Dǎng Shēn; 党参) is a major component of several Traditional Chinese Medicine (TCM) preparations, such as Fufang E’jiao Jiang, Fukeqianjin formula, and other listed in Table 1. Dangshen, which is also known as Pilose Asiabell roots or “poor man’s ginseng”, can be used to treat multiple diseases, such as idiopathic membranous nephropathy [1], or to facilitate hematopoietic stem cell transplantation [2]. The TCM preparation is commonly employed for replenishing the vital energy (Qi) deficiency, strengthening the immune system, improving gastrointestinal functions, and curing gastric ulcers. Radix Codonopsis corresponds to the roots of the plant *Codonopsis pilosula*, which is an herbaceous perennial climbing plant with a tuberous rootstock (Fig. 1).

**Table 1:** Traditional Chinese Medicinal preparations including a *Codonopsis* plant

TCM name	Plant content	Activity or use	Refs.
Dangshen	Roots of *C. pilosula*	To promote Qi. immunoregulation	[9]
Fukeqianjin formula (FKQJF)	8 Plant ingredients including roots of *C. pilosula*	Treatment of gynecological inflammatory diseases (chronic cervicitis, chronic pelvic inflammatory disease, endometritis)	[113–115]
Fufang E’jiao Jiang (FEJ)	TCM derived from “Liangyi Ointment”. Mixture of 5 plants including *C. pilosula*	Medication against weakness and anemia (used to treat chemotherapy-induced myelosuppression)	[116–118]
Shenqi Fuzheng injection (SFI)	Extract made from Radix Astragali and Radix Codonopsis	Treatment of chronic heart failure. Use as an adjunct to cancer chemotherapy treatment. Improvement of cancer-related fatigue	[119–123]
Weikang Keli	Herbal formula made from 6 plants including *C. pilosula*	Treatment of gastric cancer	[53]
Wenxin Keli	TCM containing amber and four herbs, including *C. pilosula*	Prevention and treatment of cardiac arrhythmias, including atrial and ventricular arrhythmias	[83]
Bu-Fei decoction	Consists of 6 herbal Chinese medicines including *C. pilosula*	Medication to supplement Qi. Used to alleviate lung cancer related symptoms. Immuno-stimulation	[124, 125]
Gu-Ben-Fang-Xiao decoction	Preparation made from 11 plants, including *C. pilosula*	Treatment of asthma in remission stage	[86, 126, 127]
Yi-Gan-Ning granule (YGNG)	Polyherbal preparation containing *C. pilosula*	Treatment of chronic hepatitis B	[128]
Eefooton	Liquid formula of herbal extracts containing *C. pilosula* and 4 other plants	Treatment of chronic kidney disease	[129]
Jianpi Yangzheng Xiaozheng decoction (JPYZXZ)	Complex preparation with 12 Chinese medicinal plants, including *C. pilosula*	Treatment of advanced gastric cancer	[112, 130, 131]
Bupi Yishen formula (BYF)	Extract mixture of nine herbs, including *C. pilosula*	Treatment of chronic kidney disease	[132, 133]

*Codonopsis pilosula* is commonly harvested from the wild and cultivated on a large scale for local use and trade. The cultivated plant generally shows a lower content in secondary metabolites than the wild plant [3]. However, root culture methods have been defined to increase the level of bioactive metabolites [4, 5]. The use of organic fertilizers [6] or the inoculation with growth-promoting rhizobacteria can increase markedly the content of metabolites [7]. The processing methods have a big impact on the quality of *Codonopsis Radix* and lobetyolin content [8].

**Fig. 2:**
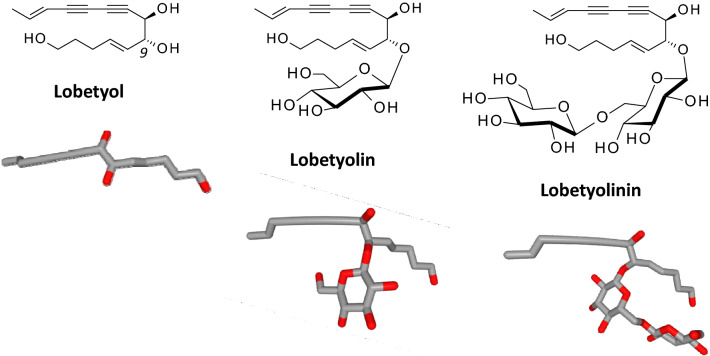
Structures of lobetyol (C_14_H_18_O_3_), lobetyolin (C_20_H_28_O_8_) and lobetyolinin (C_26_H_38_O_13_). Lobetyol is the aglycone of both glycosides. Lobetyolin (2,10-tetradecadien-4,6-diyne-8,14-diol) bears a glucose residue (β-d-*glucopyranosyl*) while lobetyolinin has a Glc-(1 → 6)-Glc disaccharide (β-d-glucopyranosyl-(1 → 6)-β-d-glucopyranoside)

The plant genus Codonopsis (Campanulaceae) contains more than 40 species, predominantly distributed in Central, East and South Asia. The best-known species are *C. pilosula*, *C. tangshen* and *C. lanceolata* which are largely used in TCM for the treatment of various symptoms and diseases including bronchitis, obesity, hepatitis, colitis, and others [9–11]. The bioactive constituents of Codonopsis extracts are very diverse, ranging from small molecules (terpenoids, saponins, alkaloids, phenolic compounds) [12] to complex polysaccharides with neuroprotective and immunomodulatory properties [13, 14]. Various anticancer compounds have been characterized from Codonopsis root extracts, notably bidesmosidic triterpenoid saponins (e.g. lancemasides A–B [15]), pyrrolidine alkaloids [16], xanthone derivatives (e.g. coxanthone B, swertiperenine [17]) and polyacetylene glycosides, such as lobetyolin. The present review is centered on lobetyolin (LBT, Fig. 2) and its pharmacological activities, with a focus on its anticancer properties.

## Lobetyolin and Related Polyacetylene Glycosides

LBT is commonly extracted from the roots of *C. pilosula* but processing the leaves into tea allows for higher levels of LBT, thus providing an appropriate and economic way of utilizing this plant reasonably [18]. LBT was found also in other plants of the Campanulaceae family, such as *Platycodon grandiflorum* [19, 20], *Lobelia* *chinensis* [21–23] and other plants listed in Table 2.

**Table 2:** Plants containing lobetyolin

Plant	Isolated compounds	Refs.
*Adenophora tetraphylla*	Lobetyolin	[134]
*Campanula alliariifolia*	Lobetyolin	[77]
*Codonopsis clematidea*	Lobetyolin, lobetyolinin	[135]
*Codonopsis tangshen*	Lobetyolin	[24]
*Codonopsis pilulosa*	Lobetyolin	[26, 27]
*Hippobroma longiflora*	Lobetyolin	[136]
*Lobelia cardinalis* (*)	Lobetyolynin, lobetyolin, lobetyol	[28]
*Lobelia chinensis* (*)	Lobetyolynin, lobetyolin, lobetyol	[29]
*Lobelia chinensis*	Lobetyolin	[21–23]
*Lobelia inflata*	Lobetyolynin, lobetyolin, lobetyol	[25, 30, 137]
*Lobelia sessilifolia* (*)	Lobetyolynin, lobetyolin, lobetyol	[31]
*Lobelia siphilitica*	Lobetyolynin, lobetyolin, lobetyol	[136, 138]
*Platycodon grandiflorum* (*)	Lobetyolynin, lobetyolin, lobetyol	[139, 140]
*Platycodon grandiflorum*	Lobetyolin	[19, 20]
*Pratia nummularia*	Lobetyolynin, lobetyolin, lobetyol	[36]
*Trachelium caerukum* (*)	Lobetyolynin, lobetyolin, lobetyol	[141]
*Wahlenbergia marginate* (*)	Lobetyolynin, lobetyolin, lobetyol	[142]

(*) hairy roots induced by *Agrobacterium rhizogenes*

Various C-14 polyacetylene glycosides have been isolated from Codonopsis species, including lobetyolin and lobetyolinin which are derivatives of lobetyol substituted with a glucopyranose and an isomaltose (6-O-β-d-glucopyranosyl-(1″ → 6′)-β-d-glucopyranoside) moiety, respectively (Fig. 2). Lobetyolin was discovered in the early 1990s from *C. pilosula* [24] and from the plant *Lobelia inflata* which is also a Campanulaceae [25]. Later, the compound was fully characterized from *C. pilosula* extracts [26, 27] and isolated from hairy roots cultures of several Lobelia species [28–32]. The induction of hairy roots by *Agrobacterium rhizogenes* is a convenient procedure to enhance considerably the production of lobetyol and lobetyolin [33].

**Fig. 3:**
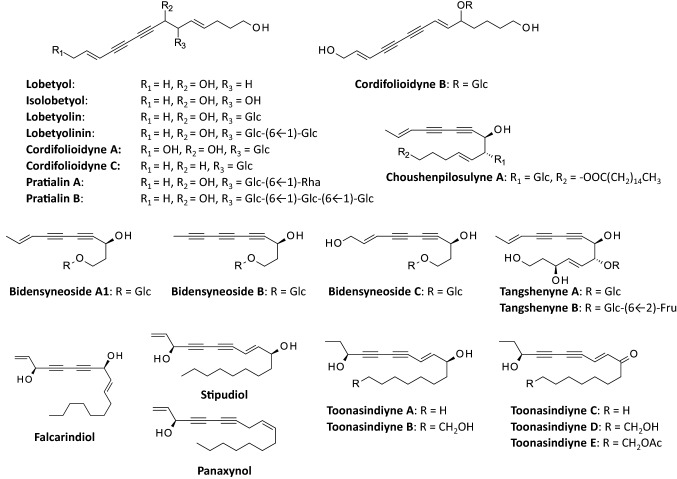
Structures of polyacetylene natural products mentioned in the review

LBT bears a structural analogy with other polyyne glycosides such as isolobetyol [34, 35], pratialins A and B from *Pratia nummularia* (Campanulaceae) [36, 37], tangshenynes A–B from *C. tangshen* Oliv. [38], and other plant products [39, 40]. In fact, *C. pilosula* contains numerous C-14 polyacetylene glycosides such as the compounds designated cordifolioidynes A–C [10, 41, 42], codonopilodiynosides A–M [43, 44] and choushenpilosulynes A–C [45]. Examples of these compounds are shown in Fig. 3. Natural polyacetylene glycosides constitute an underexplored class of compounds found in diverse plant families, such as the Campanulaceae, Apiaceae and Asteraceae families [33].

## Anticancer Activity of Codonopsis Extracts

Several types of Codonopsis extracts have shown anticancer activities. Notably a methanolic extract from *C. lanceolata* was found to inhibit cancer proliferation and to induce apoptosis of HSC-2 human oral cancer cells in vitro, with an increased expression of the protein Bak [46]. Also, a n-butanol fraction of *C. lanceolata* was found to dose-dependently inhibit the growth of HT-29 human colon cancer cells, via the production of intracellular reactive oxygen species (ROS) and polyamine depletion, leading to the induction of cell apoptosis [47]. But these anticancer effects were not specifically attributed to LBT and derivatives, but essentially to the various saponins and harman-type alkaloids found in these extracts [11]. However, in another study, an extract of *C. lanceolata* obtained by a steaming treatment was found to exhibit noticeable anticancer effects, both in vitro and in vivo, using H22 hepatocellular carcinoma cells [48]. A daily oral administration of the steamed extract at 400 mg/kg markedly reduced the tumor growth in vivo and increased the life span of mice bearing H22 tumor. An improvement of immune functions was observed together with an induction of apoptosis and inhibition of angiogenesis by the extract. This specific extract was found to contain 6 saponins (mainly lancemaside derivatives) and LBT, which may therefore contribute to the observed anticancer effects.

Another extract, from the roots of the plant *Codonopsis bulleyana* Forest ex diels (locally known as Tsoong), has been shown to display anticancer effects. This extract inhibited autophagy in cancer cells through activation of the NFκB pathway and induction of apoptosis, resulting in a dose-dependent reduction of HCT116 and SW480 colon cancer cell proliferation [49, 50]. In addition, an oral administration of a high dose (20 g/kg) of the extract reduced the growth of SW480 tumor xenografts in mice. The anticancer effect was associated with a higher level of inflammatory cell infiltration and a reduced level of tumor autophagy in vivo [51]. But recently, the anticancer activity of Tsoong was attributed, at least in part, to the flavonol aglycone isorhamnetin [52] and the phenylpropanoid cordifoliketone A [20] included in the extract. LBT may play a secondary role. Nevertheless, Tsoong seems to be an active traditional medicine for the treatment of cancer, particularly active against colon cancer [51].

**Fig. 4:**
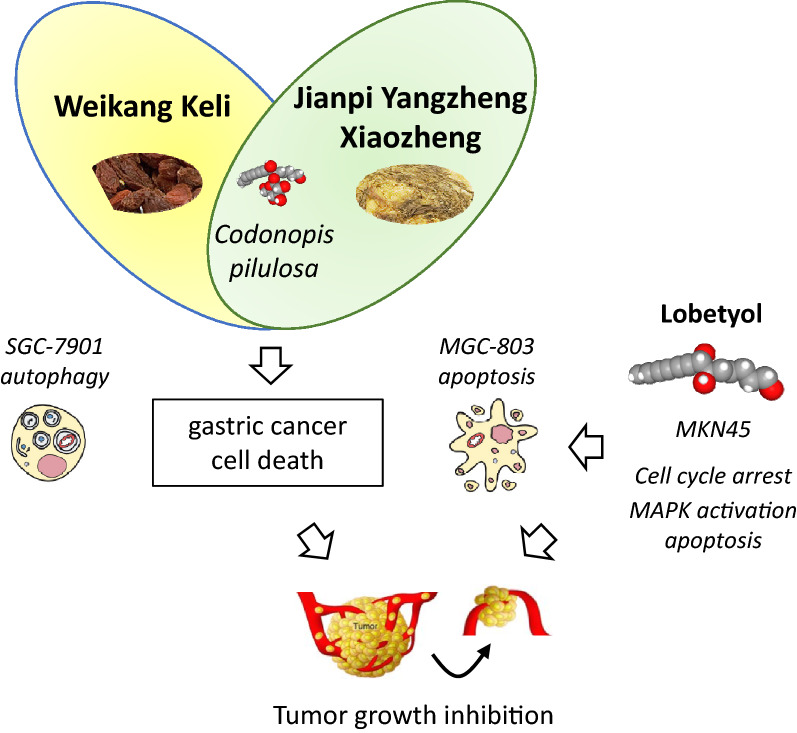
Illustration of the anticancer effects of the TCM Weikang Keli and Jianpi Yangzheng Xiaozheng, which both include roots of *C. pilosula* and have been shown to inhibit gastric cancer cell proliferation and to induce cancer cell death. Weikang Keli induces autophagy of SGC-7901 cells [15] whereas Jianpi Yangzheng Xiaozheng recipe activates apoptosis of MGC-803 cells [112]. These two medicinal preparations have demonstrated anticancer effects in vivo, reducing the growth of gastric cancer in murine models. Lobetyol was also shown to induce cell cycle arrest of gastric cancer cells and to suppress MKN45 gastric tumor growth in vivo [60]

Anticancer effects have been observed with more complex extracts derived from TCM (Fig. 4). For example, the formula called Weikang Keli (made from roots of *C. pilosula* and five other plants) was found to induce autophagic cell death of SGC-7901 gastric cancer cells and to reduce tumor growth in vivo [53], but here again the effects could not be attributed directly to LBT. Similarly, a complex phyto-preparation called Jianpi Yangzheng Xiaozheng recipe containing *C. pilosula* was found to inhibit the growth of MGC-803 gastric cancer cells in mice, with activation of apoptosis and autophagy [26] but the effect cannot be ascribed to specific compounds. This type of extract is usually well tolerated, and do not cause acute or subchronic toxicity [54]. Nevertheless, the use of Codonopsis extracts should be considered with caution because in another study, Codonopsis Radix was found to promote the development and progression of prostate cancer, via enhancement of androgen receptor expression [55].

## Anticancer Activity of Lobetyolin

LBT can be easily extracted from Radix Codonopsis, using traditional organic solvent-based methods (extraction with n-butanol in Chinese patent CN104231015A (2014) and with ethanol in Chinese patent CN106749444A (2017)) or using green chemistry procedures, such as extraction with supercritical carbon dioxide in the presence of ethanol [56]. Specific analytical methods have been developed to determine the LBT content in plant roots and extracts [20, 22, 57, 58] and to perform quality assessments of the diverse phyto-preparations [59]. The compound can be used as a bioactive marker to evaluate the quality of Radix Codonopsis preparations [12]. Synthetic methods can also be applied to obtain LBT and analogues [39] but the chemistry of these polyyne glycosides is not well developed.

Lobetyol exhibits mild cytotoxic properties but its glycoside derivative lobetyolin is more potent. The growth of PC-3 prostate cancer cells is more intensely inhibited in the presence of LBT (IC_50_ = 5.7 μM) than with lobetyol (IC_50_ = 12.7 μM) and the related compound isolobetyol (Fig. 3) is slightly less active than lobetyol (IC_50_ = 6.8 μM) [35]. Another study has confirmed the mild cytotoxic potency of lobetyol (IC_50_ = 11.7 μM and 9.6 μM, using the lung cancer cell lines MSTO-211H and NCI-H292, respectively) [34]. However, despite its lower cytotoxic potency, lobetyol has revealed interesting anticancer properties. It inhibited the growth of MKN45 human gastric cancer cells in vitro and suppressed the growth of MKN45 tumor xenografts in vivo, in a dose-dependent manner (Fig. 4). The drug induced cell cycle arrest and tumor cell apoptosis, via activation of the MAPK pathway [60].

**Fig. 5:**
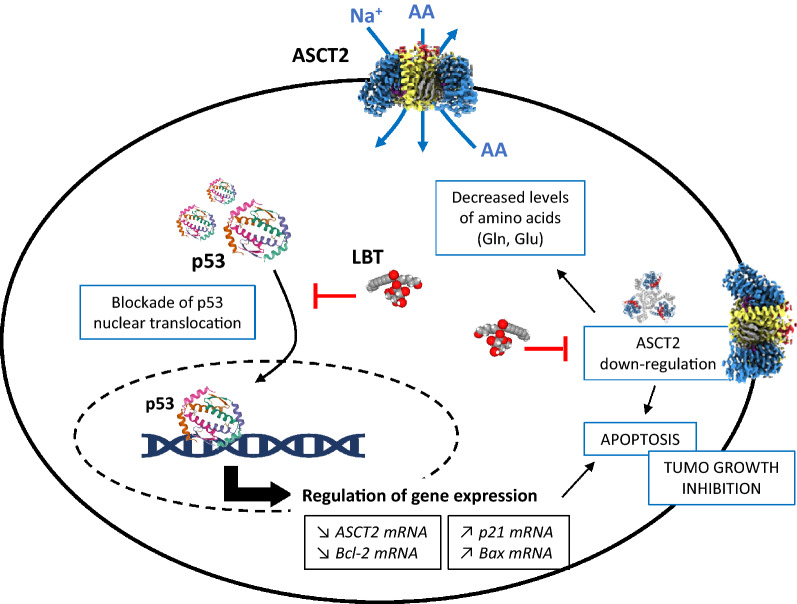
Illustration of the mechanism of action of LBT which was shown to block the transport of the tumor suppressor p53 from the cytoplasm to the nucleus, thereby modulating gene expression and inducing apoptosis of HCT-116 colon cancer cells [61]. Through this p53-dependent mechanism, the drug decreases the content of glutamine and glutamic acid, and down-regulates the expression of the amino acid transporter ASCT2 at the mRNA and proteins levels. ASCT2 (SLC1A5) is a sodium-dependent neutral amino acid antiporter involved in transmembrane traffic of glutamine which is exchanged through the cell membrane with smaller amino acids such as serine or threonine. A marked antitumor effect has been observed using HCT116 tumor xenograft upon treatment with LBT (10–40 mg/kg)

The anticancer activity of LBT apparently relies on the inhibition of glutamine metabolism, mediated by the amino acid transporter ASCT2 (Alanine-Serine-Cysteine Transporter 2) [61]. ASCT2 is often overexpressed in highly proliferative cancer cells to fulfill enhanced glutamine demand and as such, it represents an attractive target for cancer therapy [62, 63]. Lobetyolin dose-dependently (10–40 μM range) reduced the proliferation of HCT116 colon cancer cells in vitro and induced caspase-dependent apoptosis (Fig. 5). The drug-induced apoptotic progression was dependent on the activity of ASCT2 [61]. The drug blocked the transportation of the tumor suppressor protein p53 to the cell nucleus and thus modulates the expression of several genes implicated in glutamine metabolism and apoptosis of the cancer cells. A profound down-regulation of the ASCT2 gene and protein was observed, resulting in a marked antitumor effect when mice bearing subcutaneous HCT116 tumors were treated with LBT, intraperitoneally at 10–40 mg/kg [61]. The blockade of glutamine metabolism represents an interesting approach to reduce cancer cell growth, by depriving the cells of the fuel which they need to proliferate [64]. It could be particularly useful to combat aggressive cancers characterized by a large expression of ASCT2, such as advanced laryngeal and oral squamous cell carcinoma [65–67]. Such an inhibition of glutamine uptake via down-regulation of ASCT2 in cancer cells has been previously observed with established anticancer drugs, like topotecan used to treat gastric cancer [68]. Most gastric tumors also over-express ASCT2 and the targeting of glutamine metabolism (targeting of ASCT2 transporter and/or glutamine synthetase) is a valid approach to reduce tumor growth. The ASCT2 competitive inhibitor benzylserine reduced the growth of some ASCT2-expressing gastric cancer cell lines, although the drug sensitivity of the cell lines was not necessarily associated with the level of ASCT2 expression [69]. A monoclonal antibody targeting ASCT2 was found to suppress gastric cancer growth in vivo [70, 71]. Therefore, a small molecule directed against ASCT2 could be also useful to treat gastric cancer.

## Lobetyolin Analogs with Anticancer Activities

Various acetylenic compounds have been isolated from extracts of *C. pilosula*, including compounds bearing a cyclotetradecatrienynone core [43, 44] and compounds more similar to lobetyolin, such as pratialin B (lobetyol 9-O-Glc 6 ← 1 Glc 6 ← 1 Glc) [44] (Fig. 3). Pratialin B is a triglucoside derivative of LBT also found in the roots of the plant *Pratia nummularia*, which also belongs to the Campanulaceae family [36]. This type of polyacetylene phytochemicals can be useful to combat cancer. For example, a remarkable antimetastatic effect has been observed with the polyacetylene glycoside mixture designated BP-E-F1, through inhibition of the differentiation and function of myeloid-derived suppressor cells [72].

The anticancer properties of polyacetylene glycosides have not been extensively investigated. In general, these compounds cannot be considered as potent cytotoxic agents. A modest antiproliferative action has been observed in most cases, or no cytotoxic activity at all. For examples, toonasindiyne C (Fig. 3) has shown a mild cytotoxic activity against U2OS osteosarcoma cells but not against two other cancer cell lines [73]. Polyacetylene glycosides from *Vernonia scorpioides* [74, 75] and *Launaea capitata* [76] showed little or no cytotoxic activity. The mechanism of action of these compounds should be further investigated, not in terms of cytotoxic potency but as modulators of amino acid transport, with reference to the mechanism of action of LBT mentioned above. In particular, it would be useful to study the mode of action of cordifolioidyne C [19], which is structurally very close to LBT.

## Other Bioactivities of Lobetyolin: Antioxidant and Cardioprotection

Lobetyol and LBT are antioxidant compounds [77]. Recently, LBT was found to function as a potent inhibitor of xanthine oxidase (XO) both in vitro and in vivo [78]. This enzyme is responsible for the catabolism of purines and their conversion into uric acid. XO inhibitors are useful for the treatment of hyperuricemia and gout [79] but also as anticancer agents, to control the oxidative stress which is one of the major hallmarks of cancer [80]. Inhibition of NO can lead to a variety of beneficial effects beyond urate lowering, as observed with the classical XO inhibitor allopurinol which presents also anticancer properties [81]. Anticancer XO inhibitors are actively searched [82].

LBT has revealed a cardioprotective activity, with an anti-arrhythmic activity demonstrated in experimental models [83]. A link may be established between this cardioprotective effect of LBT and the capacity of the compounds named choushenpilosulynes A–C to alter lipid metabolism in cells. These polyyne compounds were found to inhibit the expression of the squalene monooxygenase gene in HepG2 cells [45]. The cardioprotective of LBT activity supports the use of the Chinese botanical drug Wenxin Keli for the treatment of arrhythmia, in particular to reduce the incidence of ventricular arrhythmia [84, 85]. An anti-inflammatory activity has also been mentioned with other LBT-containing TCM preparations [86, 87]. Several polyacetylene glycosides display anti-inflammatory properties [88–92], such as coreosides A-D which are potent inhibitors of cyclooxygenase-2 [93].

## Discussion

Dangshen/Radix Codonopsis is a TCM largely used, alone or in combination with other herbal preparations, to alleviate Qi deficiency. Several TCM formula include Radix Codonopsis (Table 1) as a natural component to support and enhance the immune system. The use of these preparations is not restricted to cancer, far from it. But the tonifying properties of Dangshen and its immune-modulatory properties are appreciated to reinforce the activity of a specific cancer treatment or to reduce unwanted side effects. Preparations made from Dangshen contain a multitude of bioactive natural products. The present review is focused on LBT but, of course, there are many other important products in Dangshen preparations.

LBT belongs to a group of polyacetylene glycosides, frequently encountered in Campanulaceae. Here, the term polyacetylene refers to the presence of one or two carbon–carbon triple (acetylenic) bond; these products are not polymers. Lobetyolinin and lobetyolin—the di- and mono-glucosides of lobetyol, respectively—are not among the most frequently studied compounds. A search in PubMed (Sept 2020) retrieved only 44 publications on LBT and 11 citing lobetyolinin, for a total of 479 publications about polyacetylene glycosides. Hence their mechanism of action remains incompletely understood. Nevertheless, the potential benefit of LBT for the treatment of cancer is emerging. The drug has revealed modest anti-inflammatory and anti-proliferative activities.

Interestingly, LBT-containing extracts and TCM (e.g. Weikang Keli), and lobetyol have shown activity against gastric cancer [60] and other polyacetylenic compounds structurally close to lobetyol (e.g. 4,6,12-tetradecatriene-8,10-diyne-1,3,14-triol [94], panaxytriol [95]) have also shown activity against gastric cancer cell lines. Efficient treatments are lacking for advanced gastric cancer [96, 97]. It would be worth to investigate further the therapeutic potential of polyacetylene glycosides in this indication.

A recent study has identified a key aspect of the mode of action of LBT, with the discovery of the capacity of the compound to inhibit glutamine metabolism and specifically, to down-regulate the amino acid transporter ASCT2, in a p53-dependent manner [61]. Human ASCT2 is a trimeric protein (also known as SLC1A5) acting as a sodium-dependent neutral amino acid antiporter. Its transport activity can be modulated by lipophilic molecules, like the antagonist V-9302 which is a potent anticancer agent [98, 99], sulfonamide/sulfonic acid esters linked to a hydrophobic group [100], and cholesterol [101]. Given the diverse functional roles of hASCT2, the blockade of this transporter can have multiple implications in human diseases, not only in cancer [62, 102, 103]. Therefore, the discovery that LBT interferes with the correct functioning of ASCT2 is important from a therapeutic perspective. It opens the door, at last, to a better understanding of the drug action and its use. However, many aspects of the drug mechanism remain to be clarified, in particular its toxicology profile, pharmacokinetic properties and metabolism.

It is likely that LBT has several molecular targets in cells. To better comprehend the anticancer activity of LBT, it would be useful to consider the following targets which have been underlined in studies with other polyacetylene compounds:

- Heat shock protein 90 (Hsp90): this molecular chaperone is a direct protein target of the related product panaxynol (Fig. 3) which binds to the N- and C-terminal ATP-binding pockets of Hsp90 and displays a potent anticancer activity [104].
- Aldehyde dehydrogenase-2 (ALDH2): the anticancer alkynol natural products falcarinol and stipudiol (Fig. 3) target ALDH2 via covalent alkylation of the enzyme active site [105].
- Breast cancer resistance protein (BCRP/ABCG2): dietary polyacetylenes like falcarinol and falcarindiol (Fig. 3) found in vegetables, have the capacity to modulate the activity of the efflux transporter BCRP. For examples, they can inhibit efflux of the anticancer drug mitoxantrone in ABCG2-overexpressing HEK293 cell [106]. These compounds show interesting cancer chemo-preventive effects, preventing inflammation and colorectal neoplastic transformation [107, 108]. They are also potent inhibitors of pancreatic cancer cell proliferation [109].
- Nitric oxide (NO): the polyacetylene glucosides bidensyneosides A1, A2, B, C have been shown to inhibit NO production in lipopolysaccharide and interferon-g activated murine macrophages [110].

The structural analogy between LBT and the polyacetylenic compounds mentioned above plaid for an analysis of the potential effects of LBT against these targets. Moreover, a comparison of the gene expression signature of 102 TCM products has indicated that LBT shares a functional gene signature with anticancer natural products like ferulic acid and artemisinin [111]. The use of network pharmacology approach would be useful to help identifying LBT drug targets.

In summary, the anticancer properties of LBT and derivatives deserve further attention. A new direction has been opened recently with the characterization of the effect of LBT on glutamine metabolism and additional anticancer targets are proposed. Polyacetylene glycosides should be further considered as antitumor compounds and components of TCM preparations.

## Abbreviations

ASCT2: Alanine-serine-cysteine transporter 2
LBT: Lobetyolin
TCM: Traditional Chinese medicine
